# Proof of Concept for a Novel Ultralight Pneumatic Telescopic Boom Fabricated via Additive Manufacturing for Space Applications

**DOI:** 10.3390/polym17233121

**Published:** 2025-11-24

**Authors:** Teodor Adrian Badea, Alexa-Andreea Crisan, Lucia Raluca Maier

**Affiliations:** Romanian Research and Development Institute for Gas Turbines COMOTI, 220D Iuliu Maniu Av., 061126 Bucharest, Romania; teodor.badea@comoti.ro (T.A.B.); raluca.maier@comoti.ro (L.R.M.)

**Keywords:** lattice structures, telescopic boom, additive manufacturing, fused deposition modeling, pneumatic

## Abstract

This study investigates the performance characteristics of a six-segment telescopic boom prototype, two meters in length, entirely fabricated through additive manufacturing using composite materials, with the aim of optimizing the system’s strength-to-weight ratio. To meet the requirements for lightweight and high-stiffness space structures, the study focuses on validating the concept from these perspectives. The current assembly prototype weighs 2154 g for a deployed length of 2 m and a stowed length of 428 mm. The pneumatic extension system demonstrated an average pressure response ranging from 0.40 bar to 1.36 bar under a payload of up to 7370 g. At this threshold, an average inclination of 5.96° and an average deployment time of 60 s were recorded. Under an increased payload of 11,345 g, the prototype exhibited an average inclination of 1.1° and an average deployment time of 30 s, with only three segments fully deployed. This limitation is attributed to the maximum allowable internal pressure of the pneumatic tube, approximately 2.2 bar, where the high frictional and binding forces between the segments exceeded the available pneumatic energy, prematurely interrupting the extension cycle. These findings confirm the applicability of additive manufacturing to complex deployable systems and establish the mechanical and pneumatic performance limits of this prototype.

## 1. Introduction

Telescopic booms, which consist of multi-body assemblies of nested beam segments, are essential components across a wide range of applications, spanning from terrestrial truck-mounted cranes to advanced aerospace systems [1]. In the context of space-based deployable structures, the design process is primarily constrained by the stringent mass and stowage volume limitations imposed by launch vehicles [2]. These constraints have stimulated the development of several innovative deployment mechanisms, among which the Roll-Out Solar Array (ROSA) stands out. ROSA employs composite slit-tube Storable Tubular Extensible Members (STEMs) to achieve a 33% reduction in structural mass and a fourfold decrease in stowed volume, enabling the deployment of a 5.4 × 1.7 m photovoltaic array capable of generating 15 kW of power [2]. Similarly, the French space agency CNES, in collaboration with COMAT, has developed a high-precision, low-power telescopic boom tailored for orbital applications. The engineering model of this system achieves a deployed length of 3.5 m with a total mass of only 2.9 kg, operating at a nominal power consumption of 1 W and peaking at 6.6 W during the mechanical interlocking phase [3,4]. Furthermore, a joint NASA–DLR initiative aims to bridge a critical technology gap for small satellite missions through the development of thin-walled composite shell booms. These booms can be stowed within a 3 U CubeSat form factor and deployed to lengths ranging from 5 to 20 m, with the objective of achieving Technology Readiness Level (TRL) 6 [5].

Recent advances in deployable structural systems have concentrated on three principal directions: modular architectures, intelligent or adaptive actuation mechanisms, and multidisciplinary design integration [6]. A representative example is the lightweight carbon fiber telescopic boom developed by the DIT Space Research Group for sounding rocket missions, which deploys a mock electromagnetic field probe to a length of 1.63 m with a deployment accuracy of ±0.5%, while accommodating the entire assembly within a compact rocket module measuring 348 mm in diameter and 220 mm in height, with a total mass below 4 kg [7]. Other noteworthy developments include the Compact Telescopic Morphing Lattice (CTML) space boom, which can be stowed within a 1 U CubeSat envelope (1000 cm^3^) and deployed to a full length of 2 m—representing a 20-fold expansion relative to its stowed configuration [8]. Additionally, the transition from traditional metallic designs to fiber-reinforced polymer (FRP) composite tape-spring booms has been extensively investigated, with both analytical and experimental results indicating that their mechanical response exhibits higher sensitivity to the overall boom length and wall thickness than to the cross-sectional radius [9,10]. Moreover, ongoing research on inflatable lattice materials has produced a novel class of ultralight structures capable of achieving specific elastic moduli up to 10^3^ times greater than those of conventional solid lattices [11], although issues related to reliable packing and post-deployment rigidization remain significant technical challenges [12].

Complementing these advances in deployable structural technologies, the Factory in Space (FIS) concept leverages additive manufacturing (AM) in combination with in situ resource utilization (ISRU) to enable the on-orbit fabrication of large-scale structures, thereby mitigating launch vehicle constraints and supporting the long-term vision of sustainable deep-space infrastructure development [13]. This paradigm is already being validated through a series of on-orbit additive manufacturing demonstrations conducted aboard the International Space Station (ISS) and is expected to undergo further testing and maturation in low Earth orbit (LEO) [14]. In parallel, pneumatic actuation principles have been successfully adapted to soft, telescopic robotic actuators, which can be integrated into hybrid robotic grippers capable of manipulating a broad spectrum of objects—from delicate components to heavy payloads—while maintaining compliance and adaptability [15].

Round lattice booms exhibit exceptional strength-to-weight efficiency owing to their tubular lattice configuration, in which intersecting steel or alloy struts form a crisscrossed framework that ensures high structural rigidity, while the circular cross-section provides uniform load distribution and enhanced resistance to global buckling. The mechanical performance of such systems is typically evaluated through key structural metrics, with the high specific stiffness and strength-to-weight ratio of PETG-CF composites enabling increased lifting capacity and extended reach without a proportional rise in boom mass or dimensions [16,17,18]. Ensuring robust buckling resistance is particularly critical for elongated booms or those operating under steep angular configurations [19]. Experimental investigations have shown that PETG-CF specimens exhibit buckling onset within the range of 1.3–1.9 kN, corresponding to displacements of approximately 0.9–1.0 mm, thereby demonstrating the capacity to sustain elevated compressive loads and accommodate notable elastic deformation prior to structural failure [20]. The dynamic response characteristics were likewise assessed to verify the boom’s ability to withstand time-dependent excitations—such as wind gusts, seismic vibrations, or payload-induced oscillations—without incurring excessive displacement or resonance effects [21,22]. In parallel, stereolithography (SLA)-based vat photopolymerization techniques have been employed to fabricate intricate gyroid and diamond lattice architectures, revealing that variations in the constituent material properties and lattice porosity exert a significant influence on the mechanical behavior, manufacturability, and volumetric precision [23].

These advancements collectively underscore an ongoing paradigm shift in the design and manufacturing of space structures, emphasizing lightweight, reconfigurable, and autonomously deployable architectures. In this context, the present study is motivated by the need to explore novel design and fabrication strategies for next-generation deployable systems, focusing specifically on the development, additive manufacturing, and experimental characterization of a pneumatically actuated telescoping lattice boom constructed from composite materials.

## 2. Design Concept

**Figure 1:**
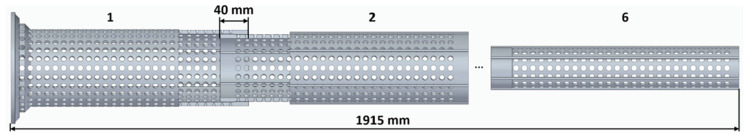
Assembly of the six additively manufactured elements of the telescopic boom prototype.

The six-element pneumatic telescopic boom prototype, measuring 1915 mm in length, features a uniform 2D lattice structure optimized to minimize the overall weight, with each element fabricated from the same composite material via additive manufacturing. The structural architecture, detailed in Figure 1, was designed in SolidEdge 2021.

The proposed design incorporates six sliding splines of 3 mm diameter and 3 mm height located between the telescope elements to prevent relative rotation between them, ensuring precise alignment during extension. This feature enables smooth, non-binding deployment, preventing lateral movement or misalignment that could compromise the boom’s structural integrity and operational reliability.

The geometric characteristics of the lattice hole patterns for each telescopic element are provided in Table 1. The repeating lattice pattern across all elements reduces the system mass, ensures structural consistency, and simplifies fabrication, providing a strong yet lightweight solution for deployable applications.

This approach enables the fabrication of a complex, multi-component structure in a single, efficient process, leveraging the advantages of additive manufacturing to produce a lightweight yet structurally robust system.

The design demonstrates a novel methodology for developing highly integrated and functional telescopic 2D lattice structures by combining advanced materials with modern manufacturing techniques to meet the severe requirements of diverse engineering applications. The structure maintains its pneumatic functionality through an inflatable tube incorporated within the lattice framework, representing the system’s fundamental operating principle.

## 3. Manufacturing

The conceptual design describes a pneumatic telescopic boom prototype manufactured using the additive manufacturing technique known as fused deposition modeling (FDM). The telescopic boom prototype was fabricated using a Prusa XL printer (Prusa Research a.s., Prague, Czech Republic) with a 1.75 mm diameter filament. The specific printing parameters are summarized in Table 2. The material chosen for the additive fabrication of the pneumatic telescopic boom is PETG-CF. PETG-CF is a PETG reinforced with short CFs of approximately 7.6 μm in a weight fraction of 20%, which has a density of 1.27 g/cm^3^ and a tensile yield strength of 52 ± 2 MPa. The material’s mechanical properties, including a tensile modulus of 1.8 ± 0.1 GPa, an elongation at the yield point of 4.5 ± 0.2%, and a flexural strength of 80 ± 2 MPa, were selected to ensure the structural integrity and performance of the lattice boom [16]. The use of carbon fiber reinforcement in the PETG matrix provides enhanced mechanical properties, such as increased stiffness and strength, which are essential for a system designed for pneumatic operation, while simultaneously ensuring an optimal balance between mechanical performance and a low coefficient of friction. This selection was based on the favorable cost-to-performance ratio of the materials and was specifically intended to achieve an optimal balance between the mechanical properties and a low coefficient of friction.

**Figure 2:**
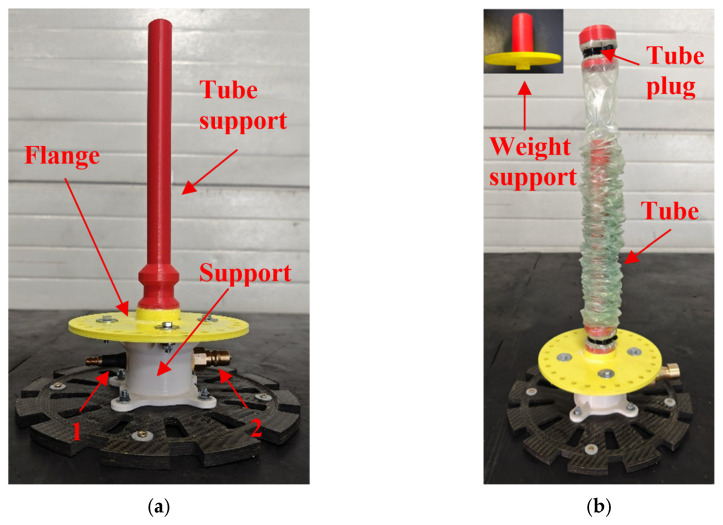
Additively manufactured components of the base support system (**a**) and components of the pneumatic system (**b**).

The pneumatic actuation mechanism, comprising the flange, support, tube support (Figure 2a) and the corresponding tube plug (Figure 2b), was fabricated using additive manufacturing. Polymaker PolyLite Acrylonitrile Butadiene Styrene (ABS) was selected to minimize the total mass of the actuation assembly, which is critical for reducing the inertial loads and enhancing the dynamic responsiveness. The carbon fiber disc at the base serves exclusively for passive mass balancing and stiffening of the support structure. It does not contribute to the pneumatic actuation but is essential for maintaining the overall stability and structural integrity under dynamic loads.

The tube support serves as the system’s support cylinder, while pneumatic actuation is provided by a flexible tubular vacuum bag (Wrightlon WL7400 SHT, Airtech, Huntington Beach, CA, USA), acting as an inflatable tube that rolls on the tube support (Figure 2b). The inflatable tube has a mass of 75 g, a wall thickness of 50 µm, an outer diameter of 41.2 ± 0.2 mm, and an active length of 1830 mm, resulting in a total volume of approximately 2.6 L, including the support enclosure. This arrangement enables controlled inflation within the actuation chamber, ensuring smooth, shock-free linear motion. The plug, a separate rigid component (red cap secured with zip ties, Figure 2b), is sealed with GS43MR (Airtech) sealant tape and zip ties. The base is sealed similarly to maintain airtight containment. During the experimental investigations, a weight support device (Figure 2b) was utilized to ensure the stable fixation of the applied masses.

Pressurized air, supplied through the quick-connect fitting 1 (Figure 2a) on the white support, is used to inflate the volume between the rigid tube support and the bag, thereby generating an expansion force on the plug that is subsequently transmitted to the payload. This configuration transforms a standard cylindrical component into a lightweight, flexible pneumatic piston system, with the quick-connect fitting functioning as the sole interface enabling precise control over the initiation of the actuation cycle. The secondary quick-connect fitting (Figure 2a) served the purpose of depressurizing the bag upon completion of the experimental procedure, thereby ensuring safe handling and system reset for subsequent tests.

The deployment kinematics of the actuation system are regulated by compressed air supplied from a Xiaomi Portable Electric Air Compressor 2 (Xiaomi Communications Co., Ltd., Beijing, China). The compressor has a maximum volumetric airflow rate of approximately 18 L/min and an adjustable pressure range up to 10.3 bar. Pressure is measured with high accuracy (±0.06 bar) and displayed with a resolution of 0.1 bar, enabling precise control of the actuation process.

## 4. Experimentation and Discussion

**Figure 3:**
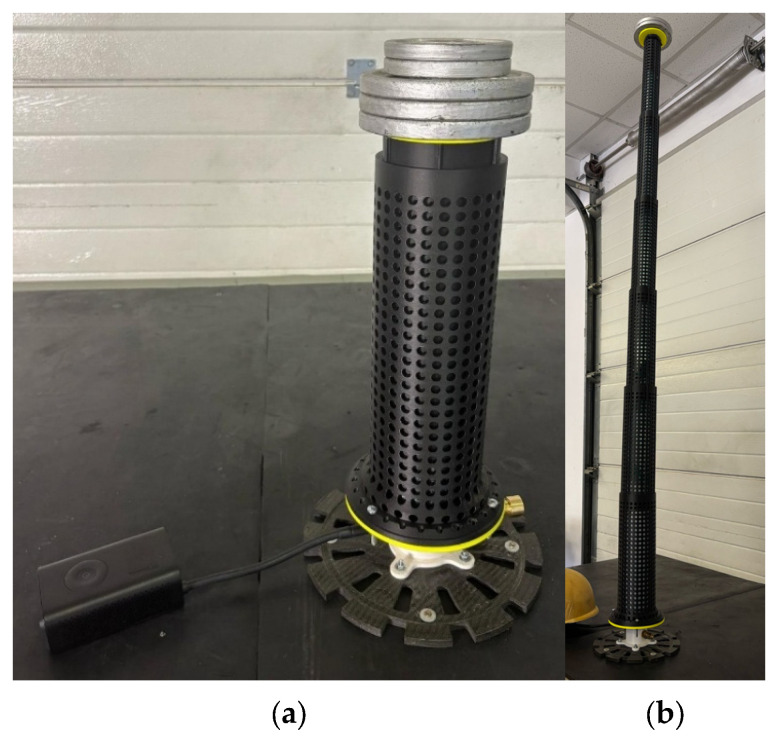
(**a**) Fully assembled additively manufactured telescopic boom prototype with pneumatic system and additional mounted payload; (**b**) Fully deployed additively manufactured telescopic boom prototype with additional mounted payload.

The six-segment pneumatically actuated telescopic boom prototype (Figure 3) was experimentally evaluated under varying payload conditions to determine the performance limits of the pneumatic actuation system. The actuation mechanism employed an inflatable tube pressurized using a Xiaomi Portable Electric Air Compressor 2 (Xiaomi, Beijing, China). As the compressor does not actively regulate pressure, inflation occurred passively, while internal pressure, deployment time, and angular deflection were systematically measured to assess the system’s operational performance and limitations.

**Figure 4:**
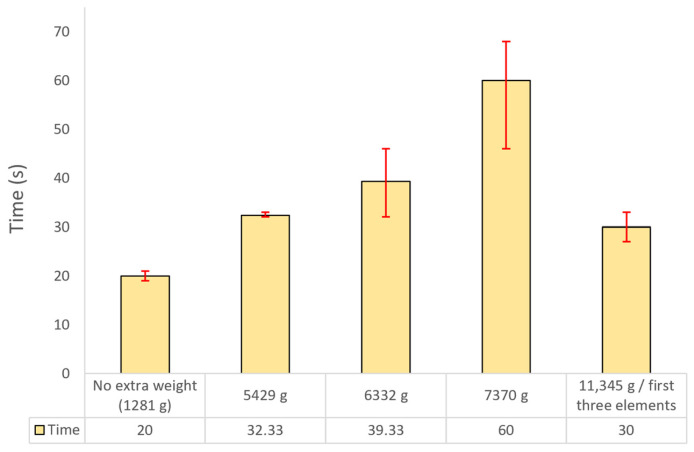
Deployment time of the telescopic boom prototype system under various payloads for maximum pressure of the pneumatic operation.

The deployment time results (Figure 4) demonstrate a clear positive correlation between the applied load and the time required for full extension, increasing from 20 s in the unloaded configuration to 60 s under a 7370 g payload. This trend reflects the progressive mechanical resistance experienced by the pneumatic actuation system, primarily governed by friction between the inflatable tube and the inner surfaces of the telescopic elements, as well as inter-element friction amplified by clearance-induced misalignment. Under the maximum applied load of 11,345 g, the deployment time decreased to 30 s, shorter than that observed at 5429 g, due to incomplete deployment. The excessive payload generated binding and static friction forces that exceeded the maximum allowable internal pressure of the pneumatic tube (approximately 2.2 bar), thereby restricting full extension and resulting in reduced angular deflection and shorter operating duration.

The pressure measurements obtained during the experimental campaign exhibit a monotonic increase as a function of the applied payload, thereby confirming the expected pneumatic response of the system. In the baseline configuration, corresponding to the unloaded state of the six-segment boom with a total mass of 1280 g—including the five telescopic elements and the tube plug attached to the inflatable bladder but excluding the base-supported components and the first lattice section of the boom, which were not considered in the initial measurements—the system achieved full extension at an actuation pressure of 0.40 bar.

**Figure 5:**
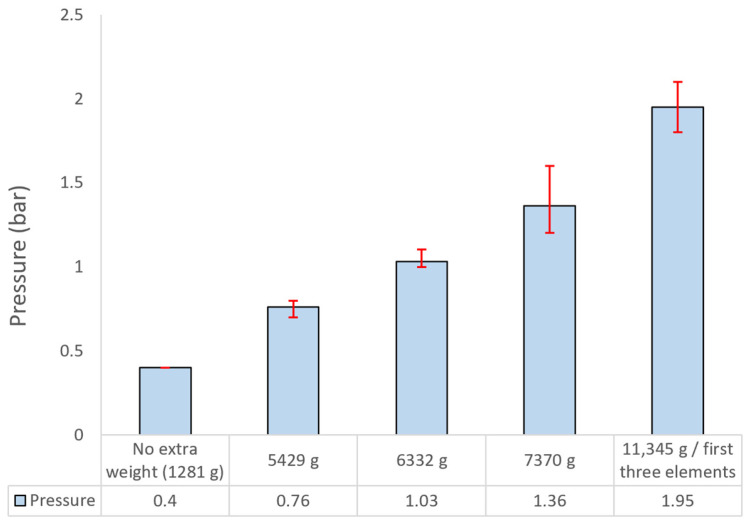
Pneumatic operation pressure of the telescopic boom prototype system under various payloads at maximum extension.

As shown in Figure 5, the incremental application of external loads resulted in a systematic increase in the required inflation pressure, reaching 0.76 bar for an additional 4 kg, 1.03 bar for 5 kg, 1.36 bar for 6 kg, and 1.95 bar for 10 kg of supplemental mass. This progression indicates an approximately linear correlation between payload magnitude and the internal pressure of the inflatable tube required for complete deployment.

The observed behavior reflects the direct influence of gravitational loading on the pressure threshold necessary to overcome both the structural stiffness of the telescopic lattice elements and the frictional interactions between the inflatable tube and the inner walls of the boom assembly. The gradual increase in actuation pressure with rising load further demonstrates the capability of the pneumatic system to adjust its operational pressure range in response to external loads while maintaining stable and controlled deployment performance. The measured inflatable tube pressures for all loading conditions are summarized in Figure 5.

**Figure 6:**
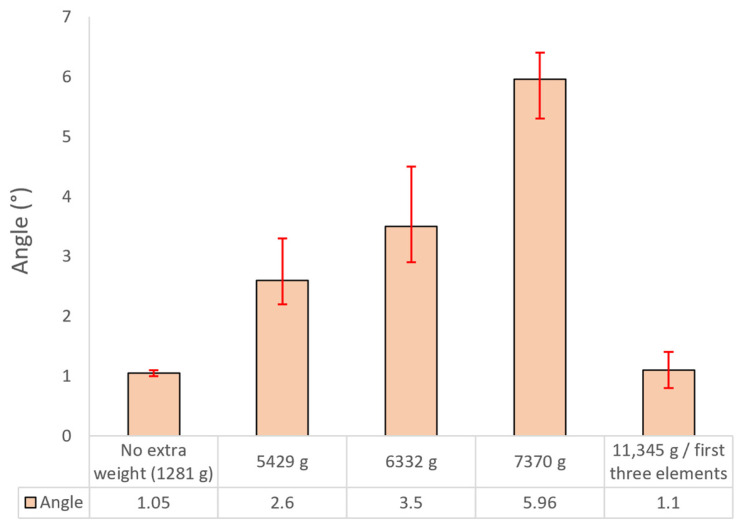
Variation in angular deflection of the telescopic boom prototype system for different payload conditions at maximum extension.

The experimental measurements of angular deflection (Figure 6) indicate a progressive increase in deflection with applied load, reaching a maximum value of 5.96° under a 7370 g payload, compared to a baseline of 1.05° recorded for the unloaded configuration (1281 g). Intermediate load cases of 5429 g and 6332 g resulted in deflections of 2.6° and 3.5°, respectively, thereby confirming the dominant influence of the bending moment on structural deformation within this operating range. Angular deflection was quantified using ImageJ software version 1.54p.

Performance was found to be strongly affected by frictional interactions, particularly between the inflatable tube and the inner surfaces of the telescopic elements. At higher payloads, both inter-element friction and clearance-induced play became more pronounced, increasing resistance during extension and requiring higher actuation pressures.

At the maximum applied load of 11,345 g, a reduced deflection of 1.1° was recorded because only the first three elements were successfully deployed, constrained by the inflatable tube’s maximum pressure capacity of 2.2 bar. The excessive mass likely induced binding and jamming between adjacent segments, thereby limiting the deployed length and shortening the effective lever arm.

Analysis of the pressure–time data recorded during the self-weight test highlights the overall resistance of the system during extension when compared to the ideal case, in which full deployment was expected within approximately 10 s, based on the known compressor performance and the total pneumatic volume of the system. The observed increase in deployment time is primarily attributed to friction between the inflatable tube and the inner surfaces of the telescopic elements, as well as inter-element friction, both of which intensify with increasing applied load. Furthermore, examination of the final angular deflection achieved after full extension, in relation to the corresponding internal pressure, demonstrates the influence of inclination angle on the effective friction coefficient of the telescopic assembly. This phenomenon increases the required actuation pressure by a factor of 2–3 in practical operation, except during the self-weight test, where the dominant resistance arises from adhesion between the inflatable tube and the inner walls. This adhesion effect diminishes progressively as the total applied load increases. The measured extension behavior further supports this observation, indicating that in all tests, both pressure and deployment time increased incrementally with each successive telescopic element, reaching up to a 25% increase for the final segment.

The telescopic boom configuration, when operated under terrestrial conditions, requires an average internal pressure of 1.36 bar to effectively deploy a payload mass of 7370 g. Based on the required gas volume of 3.536 L at 1 bar, storage at typical CubeSat tank pressures of 200–350 bar results in a minimal internal storage volume requirement. Specifically, at a storage pressure of 200 bar, the corresponding volumetric capacity is only 17.68 cm^3^, which is physically achievable using a compact high-pressure cylindrical reservoir, such as one measuring 2 cm in diameter and approximately 5.6 cm in height. These findings demonstrate the feasibility and efficiency of employing compressed inert gas (e.g., N_2_ or Ar) as an actuation medium, effectively meeting the stringent mass and volume constraints of nanosatellite platforms.

## 5. Conclusions

The study successfully validated the use of fused deposition modeling (FDM) additive manufacturing with PETG-CF composite for the fabrication of an ultralight pneumatically actuated telescopic boom, demonstrating a lifting capacity of 7370 g at an internal pressure of 1.36 bar as a promising performance benchmark for future optimization toward CubeSat applications.

The pneumatically actuated six-segment telescopic lattice boom exhibited a strong load-dependent response in both internal pressure and deployment mechanics. The required inflation pressure increased from 0.40 bar in the unloaded state to nearly 2 bar under high payloads, accompanied by proportional growth in angular deflection and extended deployment times. The results show that friction—arising from tube–wall interaction, segment misalignment, and inter-segment contact—dominates the actuation process, increasing the effective lifting pressure by a factor of 2–3 relative to theoretical predictions. At extreme loading (11.3 kg), pressure constraints and load-induced binding limited deployment to three segments, marking the onset of mechanical jamming. Overall, the findings define the operational limits and friction-driven behavior of pneumatically actuated lattice booms and provide key design guidelines for future high-performance deployable structures.

The primary limitations of the proposed concept stem from the material properties and geometric tolerances inherent to additive manufacturing, which can reduce the structural rigidity and increase the friction during extension. A potential alternative is the fabrication of lattice tubes via an industrial 3D printer or filament winding, followed by precision machining of the joints to improve segment alignment, minimize clearance-induced play, and limit excessive angular deviations during deployment. This approach is expected to enhance the structural stiffness, accommodate higher operating pressures, and increase the functional load capacity of the telescopic system.

## 6. Patents

A national patent request was filed prior to the present work in relation to the new concept ULTRALIGHT PNEUMATIC RETRACTABLE TELESCOPIC BOOM, Tedor-Adrian Badea, Raluca Condruz, reference no. RO138254A0-OSIM: 25.01.2024.

## Figures and Tables

**Table 1:** Detailed specifications of each element of the telescopic boom prototype.

Telescopic Element	Length [mm]	Outer Diameter [mm]	Wall Thickness [mm]	Lattice Holes Pattern
1	340	110	5.5	26 × 26 × Φ 8 mm
2	355	97	5	26 × 22 × Φ 8 mm
3	355	85	4.5	26 × 20 × Φ 8 mm
4	355	74	4	26 × 20 × Φ 8 mm
5	355	64	3.5	26 × 16 × Φ 8 mm
6	355	52	3	26 × 14 × Φ 8 mm

**Table 2:** Manufacturing specifications of the telescopic boom prototype and associated pneumatic actuation system.

Component	Material	Infill [%]	Wall	Temperature [°]	Nozzle [mm]	Weight [g]
Element 1	PETG-CF	25	3	265	0.4	411
Element 2	PETG-CF	25	3	265	0.4	369
Element 3	PETG-CF	25	3	265	0.4	297
Element 4	PETG-CF	25	3	265	0.4	234
Element 5	PETG-CF	25	3	265	0.4	195
Element 6	PETG-CF	25	3	265	0.4	171
Support	ABS	25	3	230	0.4	115
Flange	ABS	25	3	230	0.4	66
Tube support	ABS	25	3	230	0.4	55
Tube plug	ABS	25	3	230	0.4	15
Weight support	ABS	25	3	230	0.4	38

## Data Availability

The original contributions presented in the study are included in the article; further inquiries can be directed to the corresponding author.
